# RDM1 plays an oncogenic role in human lung adenocarcinoma cells

**DOI:** 10.1038/s41598-018-30071-y

**Published:** 2018-08-01

**Authors:** Lu Tong, Jian Liu, Wangjun Yan, Wenjiao Cao, Shihui Shen, Kun Li, Lei Li, Guoping Niu

**Affiliations:** 1 Shanghai Key Laboratory of Regulatory Biology, Institute of Biomedical Sciences, School of Life Sciences, East China Normal University, 500 Dongchuan Road, Shanghai, 200241 China; 2 Reproductive & Developmental Biology Laboratory, National Institute of Environmental Health Sciences (NIEHS), Research Triangle Prk, NC 27709 USA; 3 Department of Musculoskeletal Tumor, Shanghai Cancer Center, Fudan University, Shanghai, China; 4 International Peace Maternity and Child Health Hospital, School of Medicine, Shanghai Jiao Tong University, The China Welfare Institute, Shanghai, China; 5 The Affiliated XuZhou Hospital of Medical College of Southeast University, Xuzhou, People’s Republic of China

## Abstract

RAD52 motif containing 1 (RDM1) is involved in DNA damage repair pathway and RDM1^−/−^ cells increase sensitivity to cisplatin, a common chemotherapy drug. Lung cancer is the leading cause of cancer death worldwide. However, the role of *RDM1* in lung cancer is unknown. Here, we find that the mRNA and protein expression levels of RDM1 are significantly increased in human lung tumors, especially in lung adenocarcinoma. The lung adenocarcinoma patients with higher mRNA expression of *RDM1* show the worse clinical outcomes. Knockdown of *RDM1* in lung adenocarcinoma cells reduces cell proliferation and promotes apoptosis, consistent with the role *RDM1* in the overexpression experiments. Xenograft mouse model shows stable knockdown of *RDM1* significantly inhibits lung adenocarcinoma tumor growth. These *in vitro* and *in vivo* results conclude that *RDM1* plays an oncogenic role in human lung adenocarcinoma. Interestingly, P53/RAD51/RAD52 can be regulated by RDM1, and the negative regulation of P53 by RDM1 may be one of major mechanisms for *RDM1* to accomplish its oncogenic functions in lung adenocarcinoma. Therefore, *RDM1* may be a new target for the treatment of lung adenocarcinoma.

## Introduction

The risk of cancer can be significantly increased by disruption of genomic integrity resulted from dysfunctional DNA damage response signaling and/or aberrant activity of the key components in the DNA repair pathways. The DNA repair machineries work constantly to remove numerous DNA lesions caused by chemotherapeutic agents such as cisplatin, which contributes to drug resistance in many cancers. As resistance to standard cisplatin-based chemotherapy becomes a frequent phenomenon, cancer treatment targeting important components in the DNA repair pathways emerges to be an imminent and compelling task. RDM1 (RAD52 motif 1, or RD motif) is involved in cellular response to cisplatin, and shows similarities to RAD52, a key regulator in DNA recombination and repair, where the RD motif of RDM1 functionally resembles the N-terminal region of RAD52^1–3^. Importantly, RDM1^−/−^ cells exhibited the increased sensitivity to cisplatin^4^. More interestingly, our initial comprehensive bioinformatics exploration in multiple Oncomine expression datasets has identified RDM1 as one of the significantly up-regulated genes in human lung adenocarcinoma. Despite these discoveries, however, to date, little is known about the role of RDM1 in human cancer. Given the potential role of RDM1 in the DNA repair pathways that constitute an important aspect of cancer initiation and progression, we proposed that RDM1 might display oncogenic properties in lung cancer.

Lung cancer is a leading cause of cancer deaths, and remains one of the refractory cancer types. Lung cancer is divided into two major categories: small cell lung cancer and non-small cell lung cancer (NSCLC)^5^. Lung adenocarcinoma, one of major subtype of NSCLC, accounts for 40% of all lung cancers. The five-year survival rate of lung cancer is the lowest among the major cancers, including colon, breast, and prostate cancers^6^. Even with major clinical interventions, such as surgery, radiation therapy, chemotherapy, targeted cancer therapy, and immunotherapy, the survival rate has not been improved significantly, and lingers at only 15% within five years of treatment^7^. The clinical staging of lung cancers follows the TNM classification system, where the determining factors include: the size of the primary tumor (T), the effects on the regional lymph nodes (N), and the distant metastatic status (M). Recent years have witnessed some successes in targeted therapies for particular mutations in lung adenocarcinoma, such as those in EGFR and ALK, and these strategies have been approved for use as first-line treatment in adenocarcinoma^8–10^. Furthermore, investigation of the mutational landscape in lung adenocarcinoma can add new targets to the growing biomarker panel that may assist with the diagnosis of this cancer. As a result, it is imperative to uncover more novel molecules, which will be beneficial to the treatment and diagnosis of lung adenocarcinoma.

In this study, we found that the mRNA and protein expressions of RDM1 were up-regulated in human lung adenocarcinoma samples. Significantly, up-regulation of RDM1 mRNA level was correlated with poor clinical characteristics and risk factors, including staging, survival, recurrence, and smoking, as demonstrated by multiple Oncomine expression analyses. We knocked down and overexpressed *RDM1* in two lung adenocarcinoma cell lines, PC9 and A549, and then evaluated cancer-related phenotypes, including cell proliferation and apoptosis. We further evaluated the *in vivo* growth of the *RDM1*-knockdown cells in a mouse xenograft model. The current data support an oncogenic function of *RDM1* in human lung adenocarcinoma, supporting by the observation that RDM1 negatively affected the mRNA and protein expression of P53. Our study reveals the oncogenic function of *RDM1* in human lung adenocarcinoma.

## Results

### RDM1 is up-regulated in human lung adenocarcinoma tumors and correlated with poor clinical outcomes

**Figure 1:**
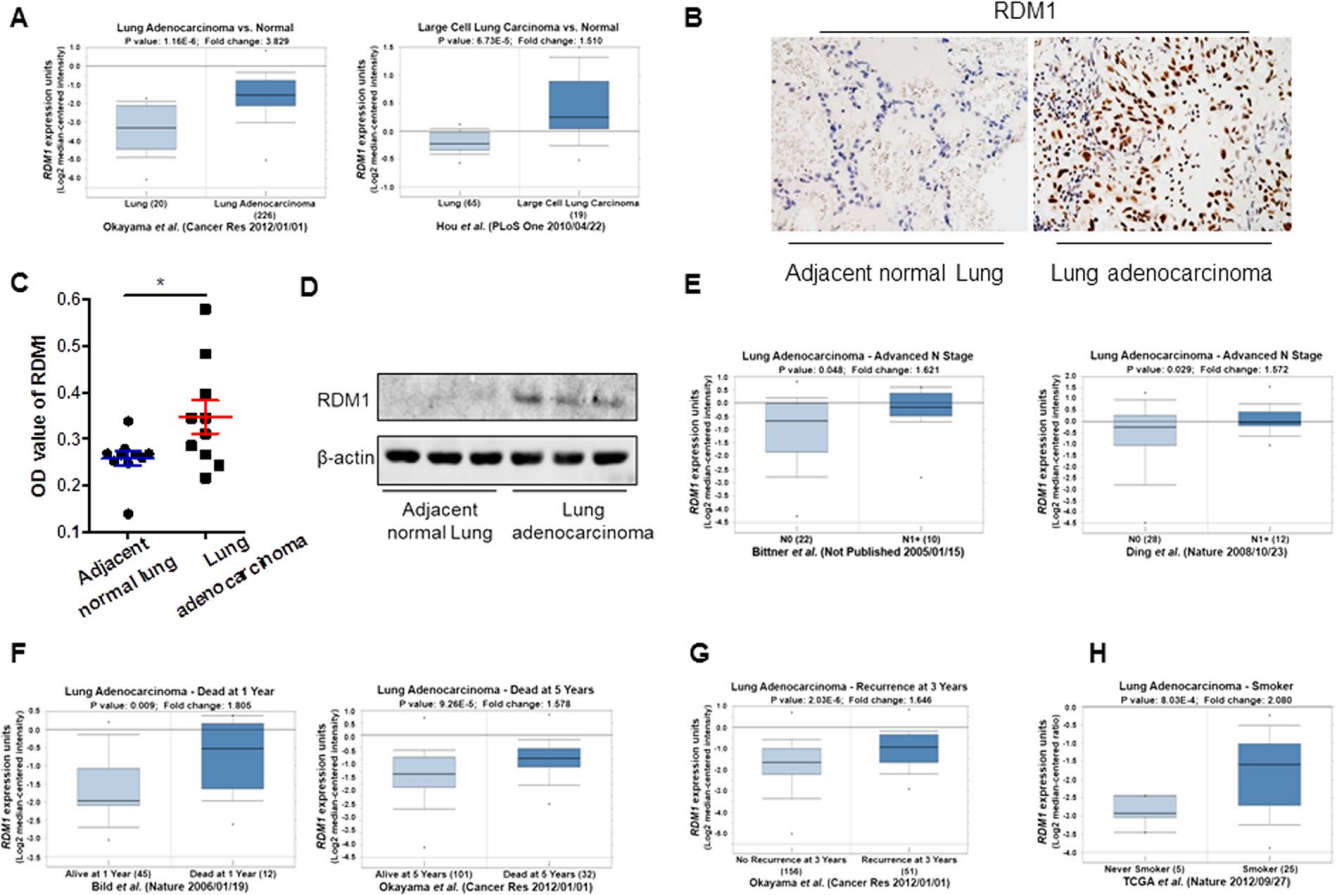
RDM1 is up-regulated in human lung adenocarcinoma tumors and correlated with poor clinical outcomes.**(A)** Oncomine box plots of RDM1 levels in human lung adenocarcinoma (left panel) or large cell lung carcinoma (right panel) and normal tissues. The data resources are listed under each plot. **(B)** Immunohistochemistry (IHC) analysis of RDM1 in clinical lung adenocarcinoma samples. **(C)** Statistical analysis of the expression of RDM1 in **B** **(D)** Western Blot analysis of analysis of RDM1 in clinical lung adenocarcinoma samples. **(E)** Oncomine box plots of RDM1 levels in advanced N stage of lung adenocarcinoma. **(F)** Oncomine box plots of RDM1 levels in lung adenocarcinoma patients deceased at 1 year or 5 years of diagnosis. **(G)** Oncomine box plots of RDM1 levels in lung adenocarcinoma patients who had recurrent cancer at 3 years or 5 years. **(H)** Oncomine box plots of RDM1 levels in smokers of lung adenocarcinoma.

Recent work has revealed high levels of RDM1 in papillary thyroid carcinoma^11^. But expression of RDM1 in lung cancer remains to be explored. We therefore performed multiple Oncomine analyses in published datasets to examine the RDM1 levels in human lung cancer with various clinical characteristics (Fig. 1)^12–15^. Interestingly, RDM1 is significantly over-expressed in lung adenocarcinoma and large cell carcinoma compared with the normal tissues (Fig. 1A). Consistent with the Oncomine results, our immunohistochemistry (IHC) and Western Blot analyses showed the protein level of RDM1 in human lung adenocarcinoma in increased compared to that of adjacent normal lungs (Fig. 1B–D).

Based on these findings, we further examined whether higher expression of RDM1 levels were positively associated with various clinical outcomes in human lung adenocarcinoma. Indeed, higher expression of RDM1 mRNA levels was found in the advanced N stage tumors (1.5 fold or higher in tumors, *p* < 0.05, Fig. 1E)^14^. Furthermore, at 1 year or 5 years of diagnosis, RDM1 levels were significantly higher in those who had deceased compared with those who were still alive (1.5 fold or higher, *p* < 0.01, Fig. 1F). Similar results were also true for patients with recurrence at 3 or 5 years (1.6 fold, *p* < 10^−5^, Fig. 1G). Therefore, in general, high RDM1 levels were correlated with poor clinical outcomes in human lung adenocarcinoma. The findings, that RDM1 was over-expressed in lung adenocarcinoma and that high RDM1 was strongly associated with poor clinical outcomes, suggesting the oncogenic role of RDM1 in human lung adenocarcinomas. Finally, we further investigated the expression patterns of *RDM1* in smokers since smoking is the leading risk factor of lung cancer. Noticeably, we observed that RDM1 was also highly expressed in lung tumors of smokers compared with nonsmokers, suggesting a positive link between smoking and the RDM1 level (2 folds, *p* < 0.001, Fig. 1H)^16^.

### RDM1 positively regulates human lung adenocarcinoma cell growth

**Figure 2:**
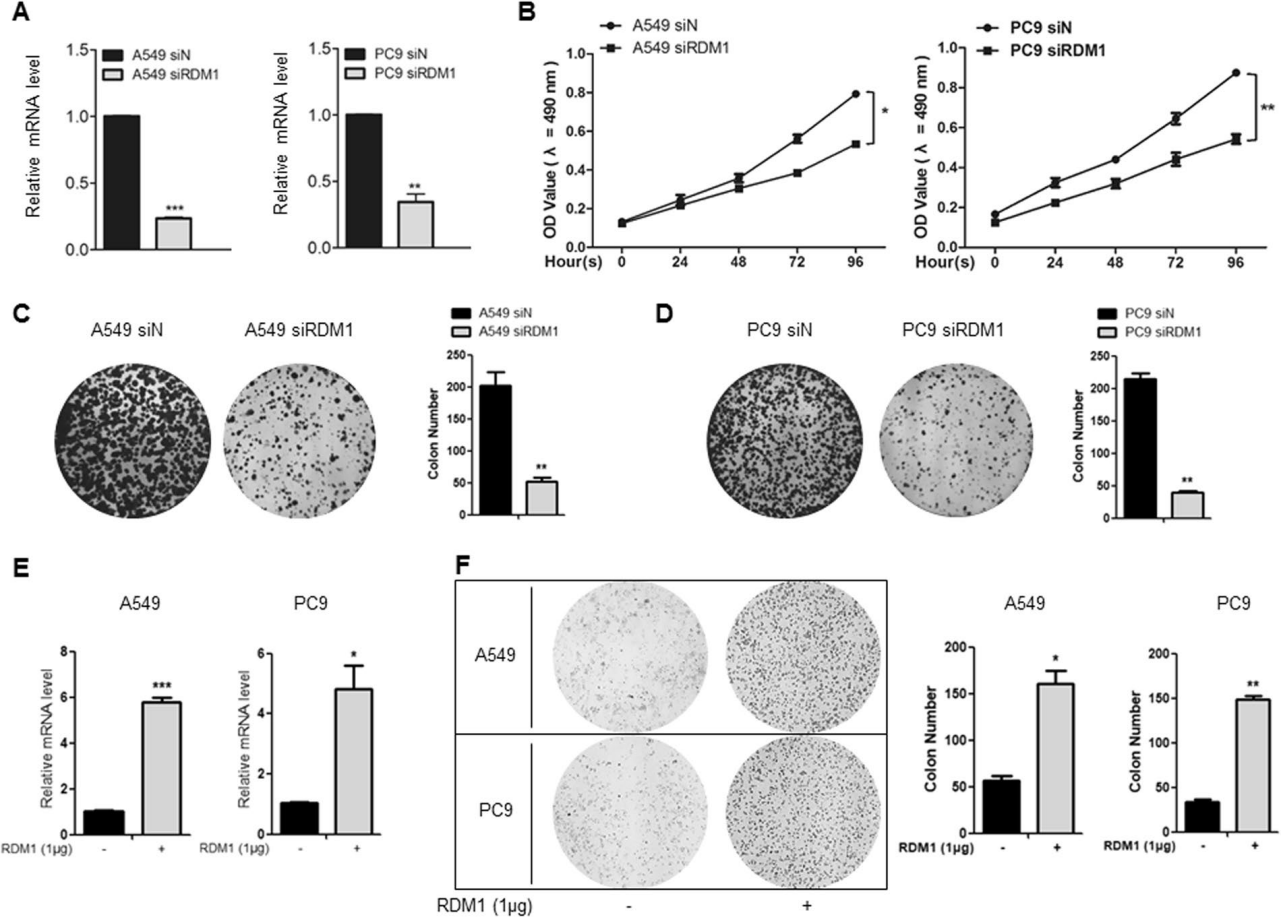
RDM1 positively regulates lung adenocarcinoma cell growth. (**A**) Knockdown efficiency of *RDM1* was evaluated by qRT-PCR in A549 and PC9 cells. (**B**) Cell growth for *RDM1*-knockdown and control cells was recorded as OD_450_ at 0, 24, 48, 72, and 96 h. (**C**,**D**) Clonogenic assay for A549 (**C**) and PC9 (**D**) cells at Day 5 after plating the cells with transfection of siRNAs. (**E**) Overexpression of *RDM1* was evaluated by qRT-PCR in A549 and PC9 cells. (**F**) Clonogenic assay for A549 cell at Day 3 after plating the cells with overexpression of RDM1.

Given that RDM1 was up-regulated in human lung adenocarcinoma, we first investigated whether RDM1 positively regulated cell proliferation. We used two lung adenocarcinoma cell lines, PC9 and A549, as our *in vitro* models. The knockdown efficiency was confirmed by qRT-PCR showing that the mRNA levels of *RDM1* were significantly inhibited after the transfection of siRNA-RDM1 (siRDM1) compared with that of siRNA-Control (siN) (Fig. 2A). It was evident that, in both cell lines, si*RDM1* cells grew significantly slower than siN at later time points (i.e. 72 or 96 h, *p* < 0.05) (Fig. 2B). These results were further confirmed by clonogenic assay at 72 h under RDM1 knockdown condition (Fig. 2C,D) or overexpression condition (Fig. 2E,F). These results concluded that RDM1 positively regulated the cell proliferation of lung adenocarcinoma.

### Knockdown of *RDM1* inhibits human lung adenocarcinoma cell growth *in vivo*

**Figure 3:**
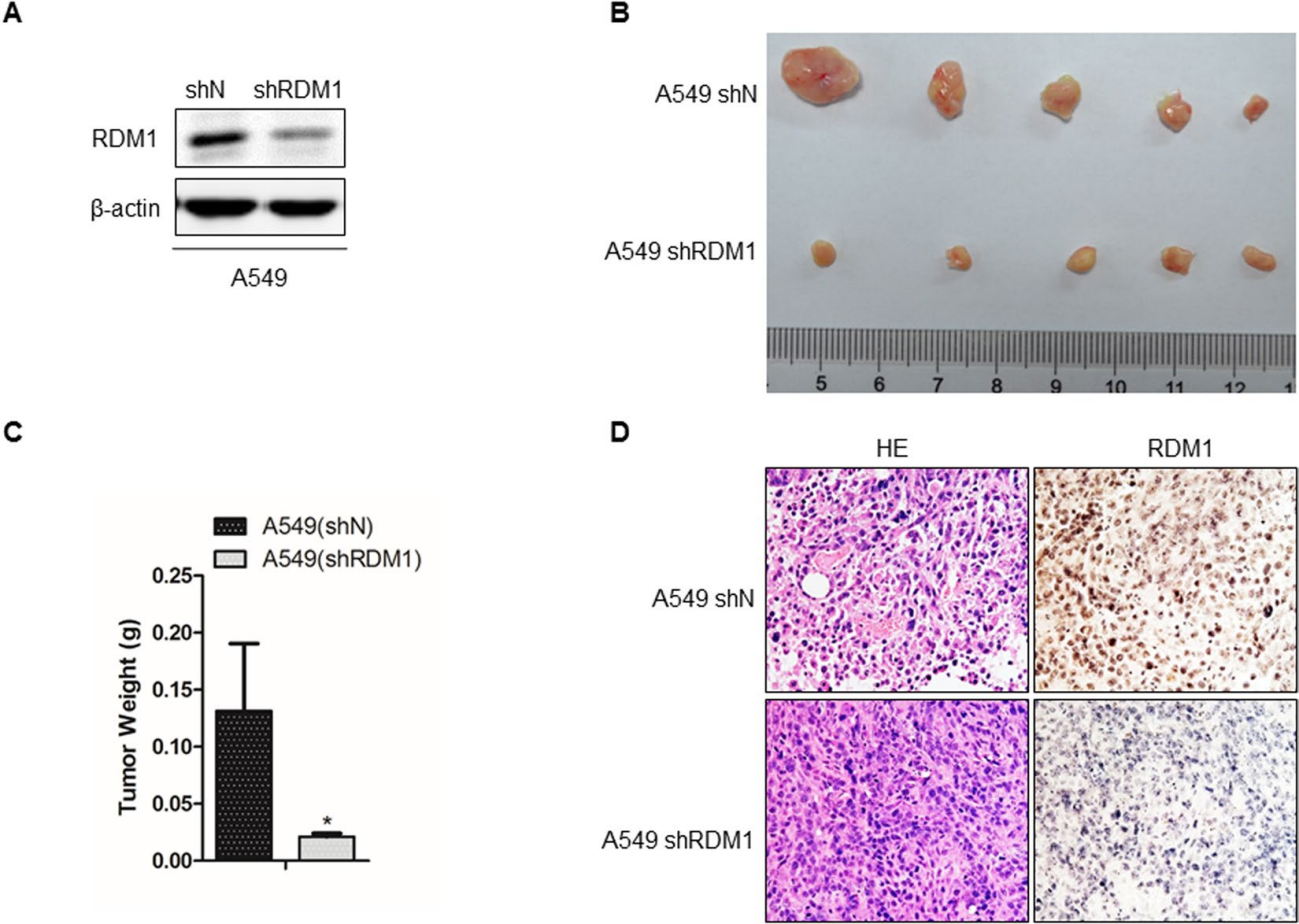
Knockdown of *RDM1* inhibits human lung adenocarcinoma cell growth *in vivo*. (**A**) Total protein was isolated from stable RDM1- knockdown (shRDM1) cell line and analyzed by immunoblotting with the antibodies against RDM1. (**B**) A549 cells with the stable *RDM1*- knockdown (sh*RDM1*) were injected subcutaneously into 6-week old immunocompromised mice. At endpoint, tumors were removed, photographed and measured. (**C**) The weights of tumors are presented as Mean ± S.D. (n = 5) *p < 0.05. (**D**) IHC staining of RDM1 was performed for *RDM1*-knockdown (shRDM1) or control (shN) tumors.

To further investigate the oncogenic role of RDM1 in lung adenocarcinoma *in vivo*, RDM1 was silenced with specific shRNA in A549 cells (A549 sh*RDM1*) (Fig. 3A). Next, we injected A549 sh*RDM1* cells into 6-week old immunocompromised nude mice and recorded the tumor sizes. At the time of sacrifice, the sh*RDM1* tumors were significantly smaller compared with the tumors of the control group, confirming the oncogenic *in vivo* role of RDM1 in lung adenocarcinoma (Fig. 3B,C). IHC staining of RDM1 protein expression was performed to confirm the knockdown efficiency of RDM1 in the sh*RDM1* tumors (Fig. 3D). Therefore, knockdown of RDM1 inhibited cell growth of lung adenocarcinoma *in vivo*.

### Knockdown of *RDM1* induces cell apoptosis in human lung adenocarcinoma cells

**Figure 4:**
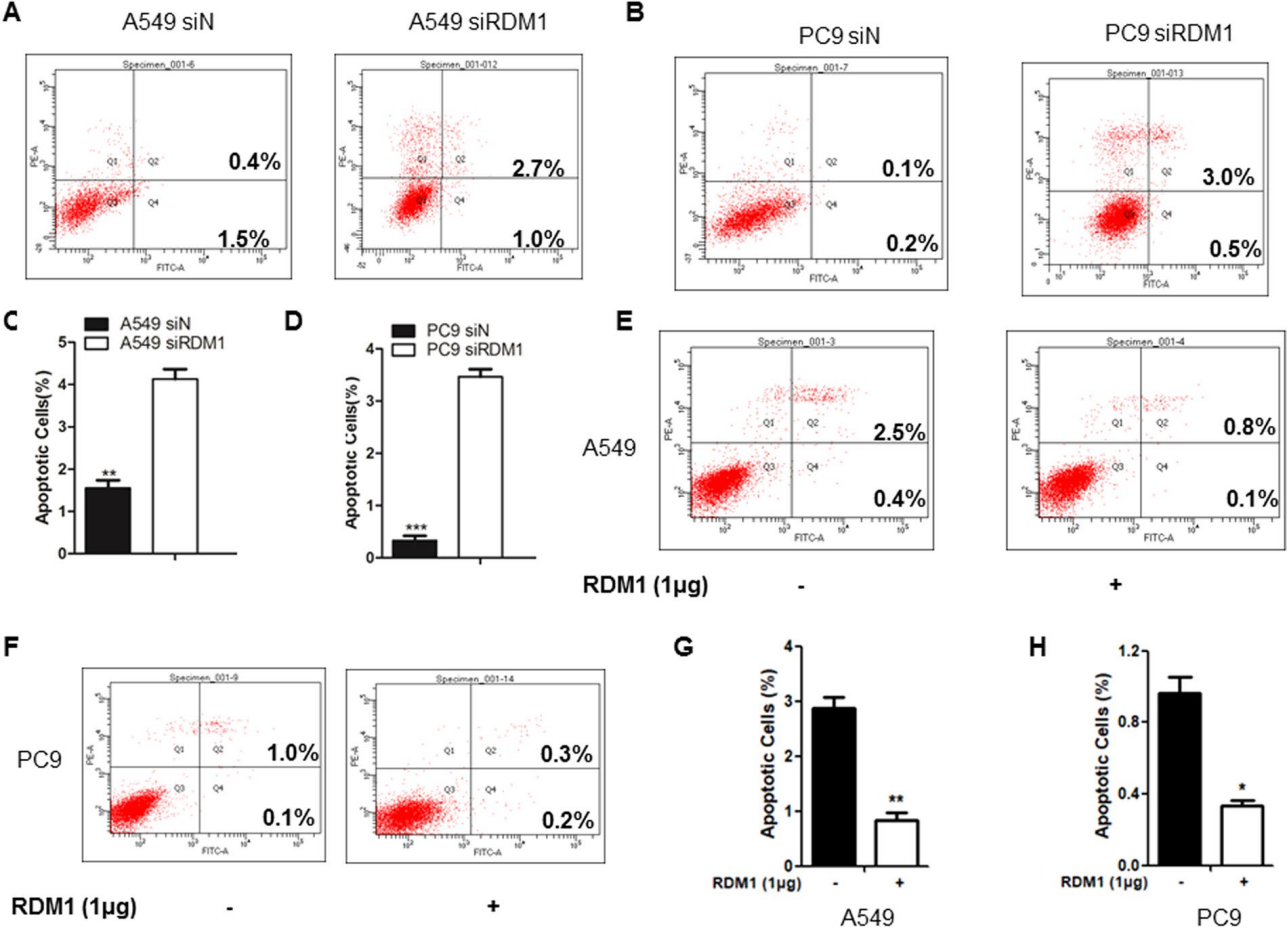
RDM1 negatively regulates cell apoptosis in human lung adenocarcinoma cells. (**A**) Flow cytometry analysis of Annexin V-FITC and Propidium Iodide (PI) double-stained populations for apoptosis in si*RDM1*A549 cells. (**B**) Flow cytometry analysis of Annexin V-FITC and PI double-stained populations for apoptosis in si*RDM1* PC9 cells. (**C**) The ratios of apoptotic cells to all cells were quantified based on three independent experiments of **A**. Data are expressed as mean ± SD (n = 3). **p < 0.01, siControl versus si*RDM1*. (**D**) The ratios of apoptotic cells to all cells were quantified based on three independent experiments of **B**. Data are expressed as mean ± SD (n = 3). **p < 0.01, siControl versus si*RDM1*. (**E**,**F**) Flow cytometry analysis of A549 (**E–G**) and PC9 (F-H) cell apoptosis after overexpression of RDM1. Similar methods were conducted as these descriptions in (**A**–**D**).

Defective growth is usually associated with dysregulated apoptosis, we therefore measured the apoptosis in the *RDM1*-kockdown cells. The flow cytometry analysis on apoptosis by double-staining of Annexin V-FITC and propidium iodide (PI) showed that si*RDM1* cells had more apoptotic populations than siN. For *siRDM1* A549 cells (Fig. 4A), more apoptotic cells (Annexin V^+^/PI^−^ and Annexin V^+^/PI^+^ combined) were detected (3.7% in knockdown cells vs. 1.9% in the control cells) (Fig. 4C). Notably, for PC9, the populations of both early stage (Annexin V^+^/PI^−^) (3.0%) and late stage apoptotic (Annexin V^+^/PI^+^) cells (0.5%) in si*RDM1* cells were higher than the control cells (Fig. 4B,D). Consistent with the results of RDM1 knockdown, overexpression of RDM1 reduced the cell apoptosis in A549 and PC cells (Fig. 4E–H). Collectively, *RDM1* in lung adenocarcinoma cells negatively regulated apoptosis, further supporting the notion that RDM1 might play an oncogenic role in lung adenocarcinoma cells.

### RDM1 regulates P53-RAD52-RAD51 in human lung adenocarcinoma cells

**Figure 5:**
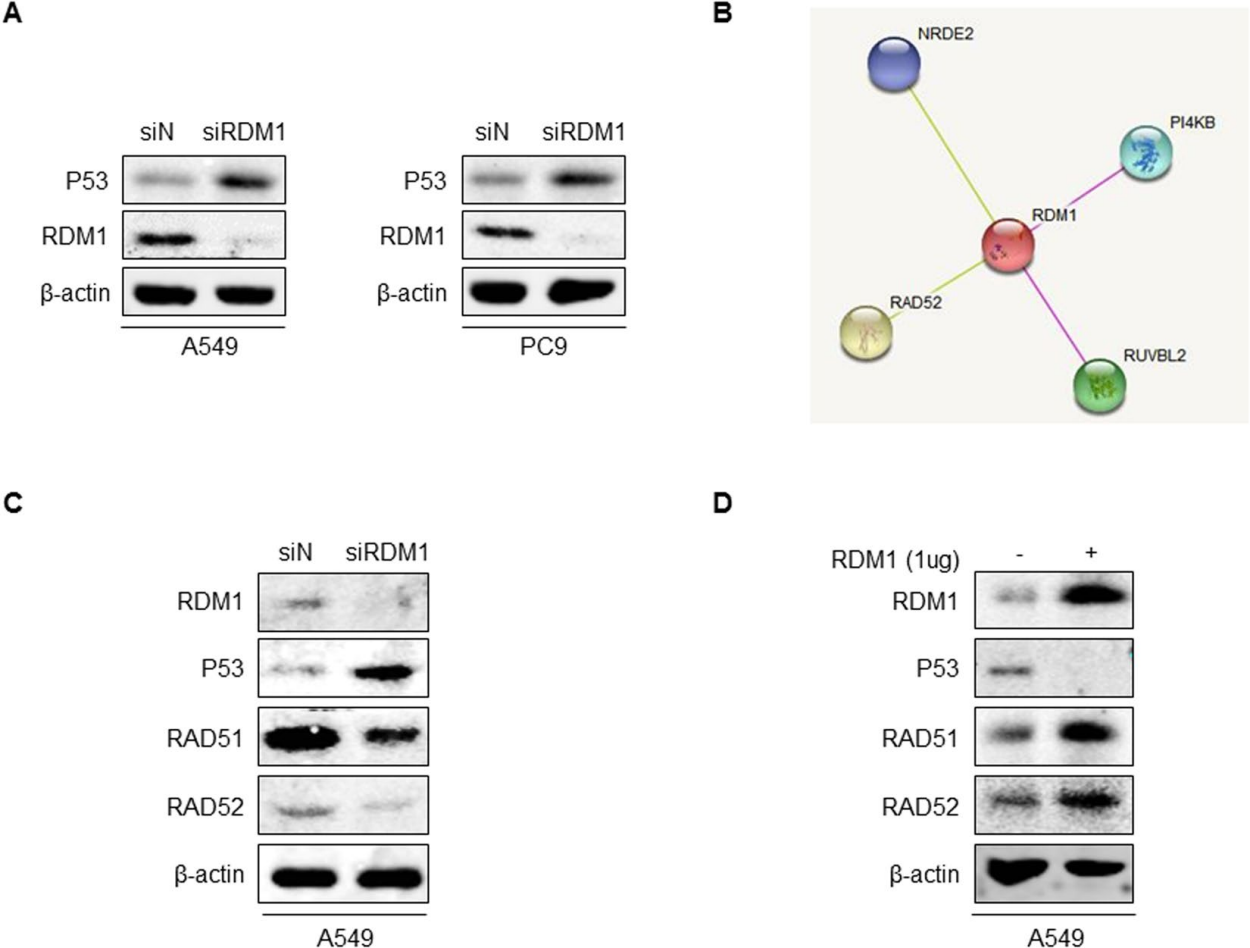
RDM1 regulates P53-RAD51-RAD52 in human lung adenocarcinoma cells. (**A**) Western blots of P53 and RDM1 in A549 si*RDM1* cells and PC9 si*RDM1* cells. (**B**) The protein interaction analysis in String datasets. (**C**) Western blots of RAD51, RAD52, P53 and RDM1 in A549 si*RDM1* cells. (**D**) Western blots of RAD51, RAD52, P53 and RDM1 in A549 cells with the overexpression of RDM1.

*TP53* is the top mutated gene in human lung cancer, including lung adenocarcinoma^16–18^. And TP53 is a central tumor suppressor and has diverse roles in DNA-repair system^19^. Given that the critical role of RDM1 in DNA damage pathway, we hypothesize that RDM1 might regulate P53 expression in A549 and PC9 cells, which are human lung adenocarcinoma cells expressing wildtype *TP53*^20^. Western blot assay showed that knockdown of *RDM1* in A549 and PC9 cells significantly increased the protein level of P53 (Fig. 5A). This results suggest the oncogenic role of RDM1 in human lung adenocarcinoma cells is partially by the negative regulation of P53. Furthermore, the analyses in String datasets indicated that RDM1 could interact with the typical DNA repair factors, such as RAD52 (Fig. 5B). It has been reported that Rad52 physically interacts with the Rad51 recombinase and serves as a mediator in the Rad51-catalyzed DNA strand exchange reaction^21^. To investigate the RDM1’s effect on these typical DNA repair factors, we knocked down or overexpressed RDM1 in A549 cells using siRNA. We found that knockdown of RDM1 decreased the protein expression of RAD51 and RAD52 with the increased protein expression of P53 (Fig. 5C–E). In sum, RDM1 regulated P53-RAD52-RAD51 in human lung adenocarcinoma cells.

### P53 negatively regulates the expression of RAD52 and RAD51 in human lung adenocarcinoma cells

**Figure 6:**
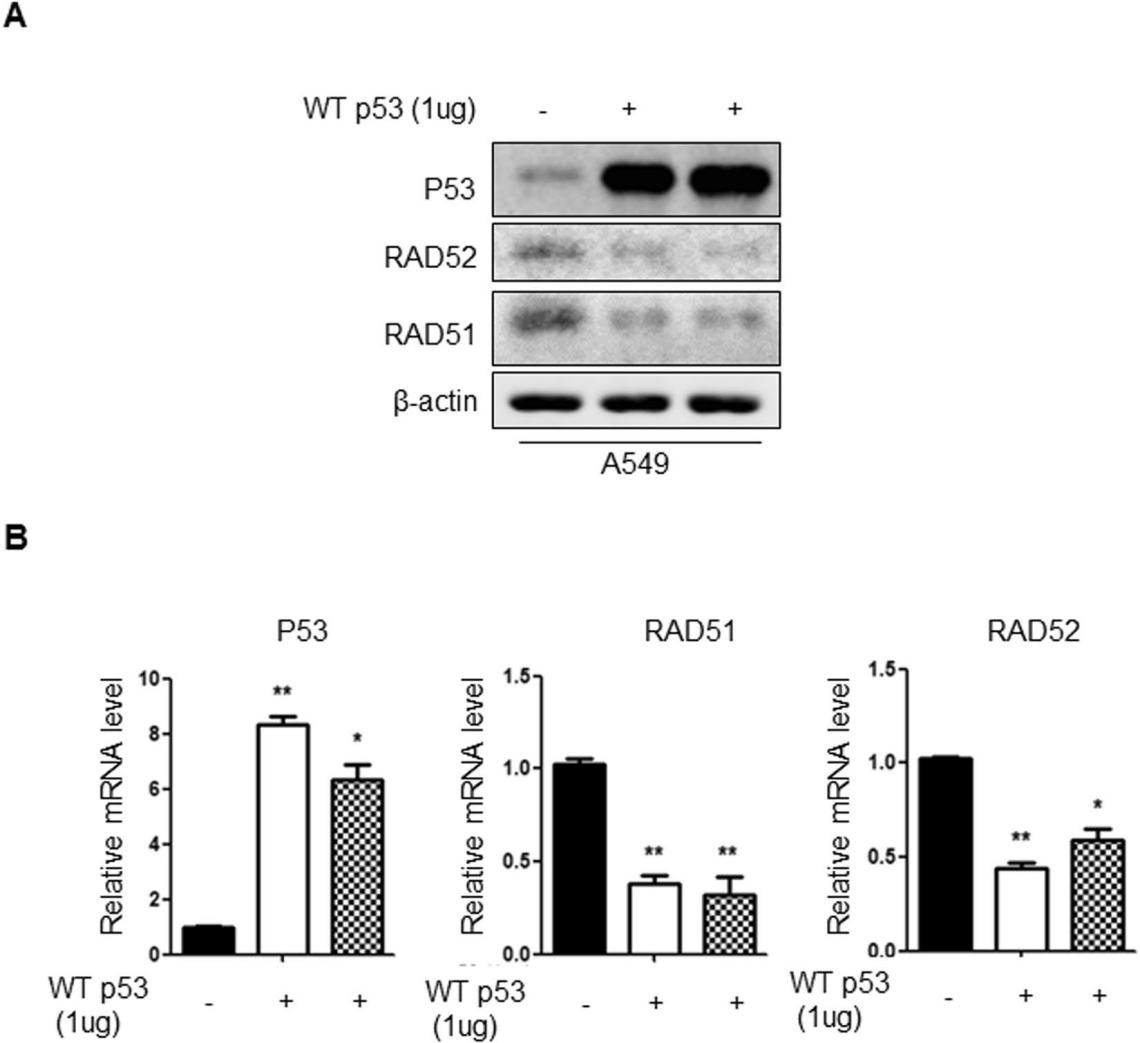
P53 negatively regulates the expression of RAD51 and RAD52 in human lung adenocarcinoma cells. (**A**) Western blots analysis of RAD51, RAD52 and P53 in A549 cells with the overexpression of P53. (**B**) qRT-PCR analysis of RAD51, RAD52 and P53 in A549 cells with the overexpression of P53.

It was reported that P53 inhibit the expression of Rad51 at transcriptional level by its specific binding to DNA^22^. Consistent with previous reports, our analyses of TP53 ChiP-Seq databases (GSM2746540, GSM1294879 and GSM2501568) show that TP53 has binding sites on RAD51 or RAD52 promoter region (Sup. Figs 1–2). Therefore, we next examined the effect of P53 on RAD51 and RAD52. The mRNA and protein expression of RAD51 and RAD52 were decreased after overexpression of P53 (Fig. 6). Taken together, these results suggested that RDM1 potentiates RAD52-RAD51 signaling via P53-mediated transcriptional suppression.

### RDM1 regulates the mRNA expression and protein stability of P53 in human lung adenocarcinoma cells

**Figure 7:**
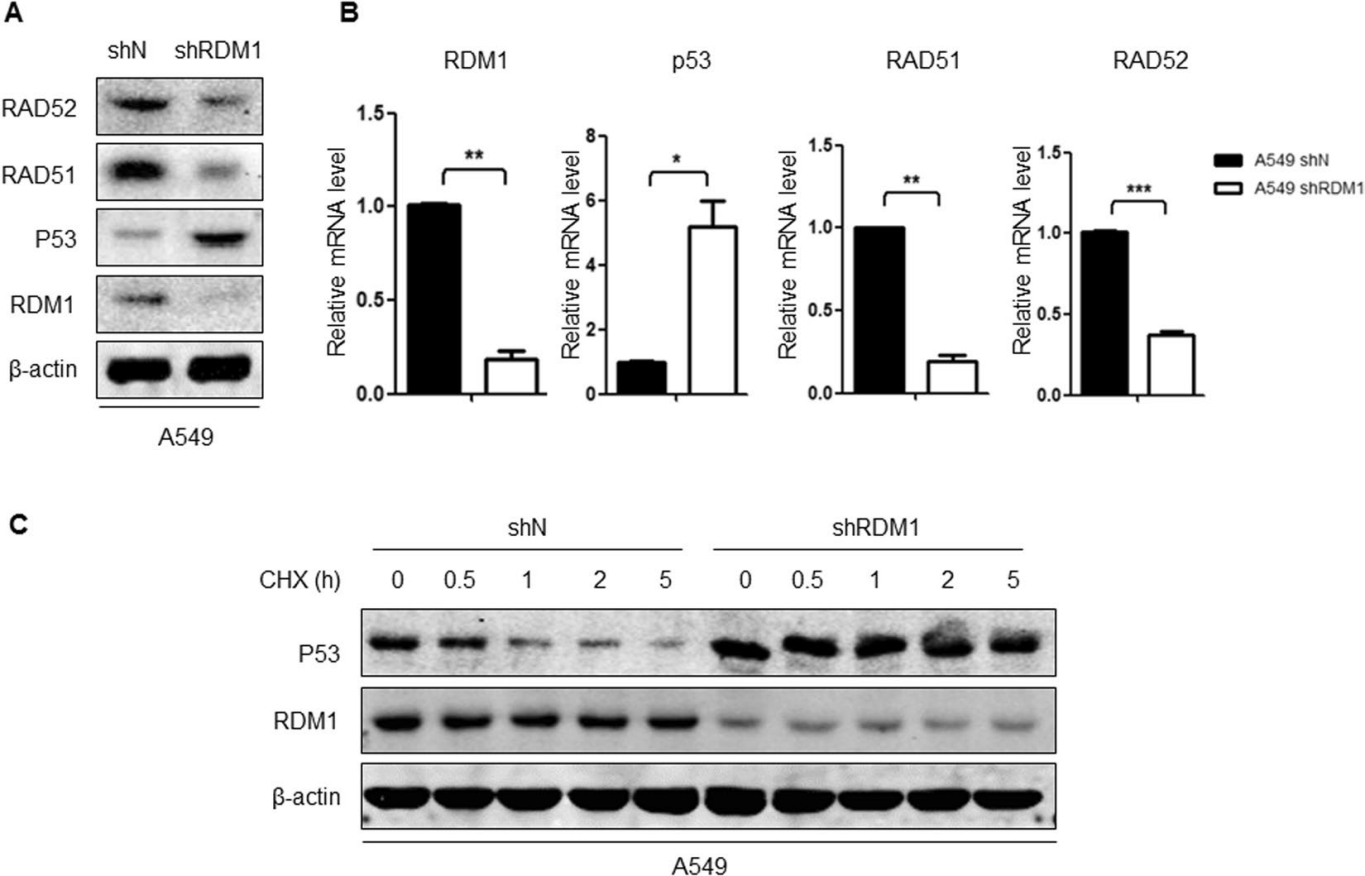
RDM1 regulates mRNA expression and protein stability of P53 in human lung adenocarcinoma cells. (**A**,**B**) Western blots (**A**) and qRT-PCR (**B**) analyses of RAD51, RAD52, P53 and RDM1 in A549 cells after knockdown of RDM1. (**C**) Western blots analysis of RDM1 and P53 in A549 cells after knockdown of RDM1 with Cycloheximide (CHX) treatment.

Given that the dysregulation of P53 may be partially responsible for the oncogenic mechanism of RDM1 in lung adenocarcinoma cells, we next examined the potential regulation P53 by RDM1. First, we employed shRNA system to confirmed the regulation of RDM1 on P53-RAD52-RAD51 to exclude the potential off-target of siRNA. Consistent with the siRNA results, knockdown of RDM1 increased the mRNA and protein expression of P53-RAD52-RAD51 (Fig. 7A,B). Meanwhile, the expression of P53 protein was more stable after knockdown of RDM1 (Fig. 7C). These results suggested P53 could be regulated by RDM1 at the transcriptional level and through protein modification.

## Discussion

In this study, we found that RDM1 played an oncogenic role in human lung adenocarcinoma cells. We observed that the mRNA and protein expression levels of RDM1 was up-regulated in human lung adenocarcinoma and correlated with poor clinical outcomes. RDM1 played an oncogenic role in human lung adenocarcinoma cells, which may be partially by inhibition of P53. Therefore, our study provides a new potential target for the treatment of lung cancer.

It has been proposed that RDM1 and RAD52 share similar functions in DNA double strand break repair and homologous recombination. Yet its function in cancer-related pathways is seldom explored. A recent work was shown that high RDM1 level was found in papillary thyroid carcinoma^11^. But the role of RDM1 in human lung adenocarcinoma remains unknown. In this study, we identified RDM1 as an oncogenic target in lung adenocarcinoma. First of all, analyses of human lung cancer samples had shown that *RDM1* was over-expressed in lung adenocarcinoma tumors, initializing the hypothesis that RDM1 may impose an oncogenic function in lung cancer. Second, knockdown of *RDM1* could reduce cell proliferation of lung adenocarcinoma cells, which might be ascribed to the increased apoptosis. Similar role of RDM1 was confirmed in the overexpression experiments. These results served as direct evidences that RDM1 might benefit for cancer cell survival. Consistent with the *in vitro* results, the *in vivo* growth of the *RDM1*-defective cells was significantly inhibited. These findings provided an important link between the chemotherapeutic resistance and the oncogenic functionality in lung adenocarcinoma. As a previous study indicated that RDM1 might play a secondary role in double-strand DNA break (DSB) repair and homologous recombination, despite that the *RDM1*^−/−^ cells exhibited elevated sensitivity to cisplatin^4^, its function as an oncogenic protein in lung adenocarcinoma became more prominent. Our discovery also restated the notion that dysregulation of the DNA damage response (DDR) led to a predisposition to cancer^23^. Since it is conceivable that disruption of the DDR signaling may affect the response to DNA-damaging anticancer therapy (such as cisplatin-based ones), future work should be focused on testing whether and how DDR pathways will be altered upon treatment of cisplatin in the context of manipulating RDM1 functionality in lung adenocarcinoma cells.

Another interesting finding of our study was that RDM1 might regulate the expression of P53, as *RDM1*-knockdown cells showed the significant upregulation of P53 protein. TP53 not only is top mutated gene in human lung adenocarcinoma^24^, but also plays a central role in regulating cell growth and apoptosis^25^. P53 regulates various downstream targets, and triggers growth arrest or apoptosis^19^. Of the DNA damage that initiates a P53 response, the molecular mechanisms by which P53 is activated following DNA double strand breaks are the most comprehensively understood^19^. In our study, the finding that RDM1 regulates P53 expression can partially explain these phenotypes observed in RDM1 silencing cells. However, whether regulation of P53 by RDM1 is the only major mechanism contributing to the decreased cell growth in *RDM1* knockdown cells needs further study. Meanwhile, how P53 regulated by RDM1 remains a question. TP53 can be regulated at different levels, including the transcriptional, epigenetic or post-translational levels^18,26^. Our data (Fig. 7) suggest that the inside mechanism related to transcriptional and post-translational levels. Therefore, following studies can examine the potential mechanisms from these two aspects.

Finally, as our Oncomine analyses have shown that up-regulation of *RDM1* is generally correlated with poor clinical outcomes, we propose that RDM1 can be a potential prognostic marker for lung adenocarcinoma. Specifically, high RDM1 levels are significantly associated with advanced lung adenocarcinoma tumors, tumors from smokers, and patients of poor outcomes (i.e. short survivals and increased recurrence). As it has been proposed that combinatorial marker panel will out-perform any single marker for prognostic predictions^27,28^, we propose that RDM1 can serve as an additional marker to the marker panel for the diagnosis and/or stratification for clinical management of lung adenocarcinoma.

In summary, our study reveals the oncogenic function of RDM1 in lung adenocarcinoma. In light of the importance of chemotherapeutic resistance and DDR pathways, future work should address the following key questions: (1) whether and how does RDM1 in DNA damage repair is related to its oncogenic functions in lung adenocarcinoma; (2) how does RDM1 regulate P53; and (3) can RDM1 be targeted for alleviating cisplatin-resistance in the therapy of lung adenocarcinoma? Answers to these questions may offer new opportunities for diagnosing, treating, and managing this major subtype of NSCLC.

### Materials

#### Animals

Six-week old mice were purchased from Shanghai SLAC Laboratory Animal CO. LTD. Mice were bred in the Animal Core Facility by following procedures ap-proved by the East China Normal University of Institutional Animal Care and Use Committee. Rules of animal welfare were applied throughout the experiment.

### Cell culture

Human non-small cell lung adenocarcinoma cell lines PC9 and A549 were obtained from ATCC. Cells were cultured in RPMI 1640 medium supplemented with 2 mM L-glutamine, 10% fetal bovine serum (FBS), 100 U/ml penicillin, and 100 mg/ml streptomycin. Cell cultures were maintained in 37 °C and humidified atmosphere consisting 5% CO_2_.

### Clinical tumor samples

Lung adenocarcinoma tumors were collected from Shanghai Cancer Center from October 2016 to July 2017. The patients were selected according to the following criteria: (1) all patients were diagnosed and confirmed by pathology; (2) patients with NSCLC were at early stages (Stage I and II) according the clinical staging method and had no other cancers; and (3) No preoperative chemotherapy or radiotherapy was administered to the cancer patients included in this study.

All samples were collected in accordance with ethical guidelines, and written informed consent was received. All patients were approached based on approved ethical guidelines, and patients who agreed to participate in this study were required to sign consent forms before being included in the study. All experimental protocols and methods were approved by Medical ethical committee of Shanghai Cancer Center (No. 2017-01-025). We also confirmed that all methods were performed in accordance with the relevant guidelines and regulations.

### RNA interference of RDM1

RDM1 siRNA sequences is 5′-GCACCAGACAUAAGGCAGUTT-3′ and RDM1 shRNA sequence is 5′-GCGAAUUACUACUUUGGUUTT-3′. The specificity of *RDM1* siRNA or shRNA sequences was confirmed by BLAST search against the human genome database. siControl-RNA was purchased from the GenePharma company.

### Overexpression of RDM1

pcDNA3.1 vector was used to express human RDM1 cDNA. Lipofectamine 2000 (Thermo Fisher Scientific) was employed to transfect 1ug pcDNA3.1-RDM1 or pcDNA3.1-vector into A549 and PC9 cells.

### Quantitative real-time PCR

Quantitative Real-time PCR using SYBR green reagents was performed as previously described^29^. The primer sequence for these detected genes: RDM1 forward: 5′-GCCCATCCTGGTTTCTATGCCC-3′; RDM1 reverse: 5′-AGACGAACCTTGACTGGAGAT-3′; RAD51forward: 5′- CAGTGATGTCCTGGATAATGTAGC-3′; RAD51 reverse: 5′-TTACCACTGCTACACCAAACTCAT-3′; RAD52 forward: 5′- TCAAGTACCGCGTGAAACCA-3′; RAD52 reverse: 5′- CGATCTTTGTTGCGGAACGG-3′; P53 forward: 5′- GGCAGACTTTTCGCCACAG-3′; P53 reverse: 5′- CAGGCACAAACACGAACCTC-3′; β-actin forward: 5′- CGTCATACTCCTGCTTGCTG-3′; β-actin reverse: 5′- GTACGCCAACACAGTGCTG-3′.

### Proliferation assay

Cell proliferation assay was performed based on a colorimetric assay system (Cell Counting Kit-8, Dojindo Molecular Technologies, Inc., USA). Both targeted-knockdown (si*RDM1*) and control cells were seeded at a density of 1 × 10^5^ cells/well before assay. At each time point, the absorbance at 450 nm was measured according to manufacturer’s instructions.

### Analysis of apoptosis

Double-staining of Annexin V-FITC-Propidium iodide (PI) was used for analyzing apoptosis. Briefly, following collection and wash, cells were resuspended by cold PBS at a density of 1 × 10^6^ cells/mL. PI (final concentration 100 µg/mL) and Annexin V-Alexa Fluor488 conjugate were mixed and added into the cell suspensions, followed by incubation at room temperature for 15 min. Flow cytometry was used to analyze apoptosis with the parameters of 494/518 nm set for Annexin V channel and 535/617 nm for PI.

### Mouse xenograft tumor model

Sh*RDM1* and control cells were suspended in cold PBS (5 × 10^5^ cells/100 µL) and injected into the mice subcutaneously on both flanks. Mice were continuously monitored for 6 weeks so the tumor size and weight were recorded. End-point mice were euthanized by CO_2_ asphyxiation, followed by removal of tumors. IHC was performed after the samples were embedded in paraffin, sectioned, and stained with hematoxylin and eosin (H&E) or IHC antibodies.

### Western blot

For Western blot, proteins were separated on 12% SDS-polyacrylamide gels and transferred to polyvinylidene difluoride (PVDF) membranes. Membranes were blocked in 5% non-fat milk in Tris-buffered saline (plus 0.1% Tween-20). RDM1 (proteintech, 20156-1-AP), p53 (santa cruz, sc-6243), RAD51 (abcam, ab133534), RAD52 (abcam, ab124971) and β-actin (MBL, M177) antibodies were purchased. Blots were detected by chemiluminescence and exposed on X-ray films.

### Immunohistochemistry (IHC)

IHC was performed for continuous sections from paraffin-embedded blocks. Antigen retrieval was performed by microwaving for 3 min in citrate-buffered solution (pH 6.0). Blocking was done by incubation with 10% goat serum at room temperature for 30 min. Sections were incubated with RDM1 (proteintech, 20156-1-AP) antibody that are indicated in this study for overnight at 4 °C. Staining with secondary antibody (conjugated with horseradish peroxidase) was performed for 1 hr at room temperature. Sections were finally stained with 3, 3- diaminobenzidine tetrahydrochloride (DAB) and counterstained with hematoxylin. Normal rabbit serum was used as negative control in place of the primary antibody. The IHC results are quantified using ImageJ (fiji-win64) as previously described^29^.

### Statistical analysis

Student’s *t* tests were performed to obtain the statistical significance. A *P* value < 0.05 was considered significant.

## Electronic supplementary material

supporting information
