# Supplementary material for: RDM1 plays an oncogenic role in human lung adenocarcinoma cells
Source: Sci Rep. 2018 Aug 1;8:11525. doi: 10.1038/s41598-018-30071-y (PMC6070564; doi:10.1038/s41598-018-30071-y)
Supplement: Supplementary file 1 — supporting information [file 41598_2018_30071_MOESM1_ESM.pdf]

a title page

RDM1 plays an oncogenic role in human lung adenocarcinoma cells

Lu Tong[1*], Jian Liu[2*], Wangjun Yan[3*], Wenjiao Cao[4*], Shihui Shen[1], Kun Li[1], Lei Li[1] and Guoping Niu[5‡]

[1] Shanghai Key Laboratory of Regulatory Biology, Institute of Biomedical Sciences, School of Life Sciences, East China Normal University, 500 Dongchuan Road, Shanghai 200241, China.

[2] Reproductive & Developmental Biology Laboratory, National Institute of Environmental Health Sciences (NIEHS), Research Triangle Prk , NC 27709, USA.

[3] Department of Musculoskeletal Tumor, Shanghai Cancer Center, Fudan University, Shanghai, China.

[4] International Peace Maternity and Child Health Hospital, School of Medicine, Shanghai Jiao Tong University, The China Welfare Institute, Shanghai, China.

[5] The Affiliated XuZhou Hospital of Medical College of Southeast University, Xuzhou, People's Republic of China.

*These authors contributed equally to this work

Address correspondence to:

Guoping Niu

Department of Clinical Laboratory, Affiliated to Medical College of Southeast University and Xuzhou Central Hospital

Email: gp_niu15@163.com

Sup. Figure 1 for Figure 6

ChiP-Seq analysis show that p53 has binding sites on RAD51 promoter region.

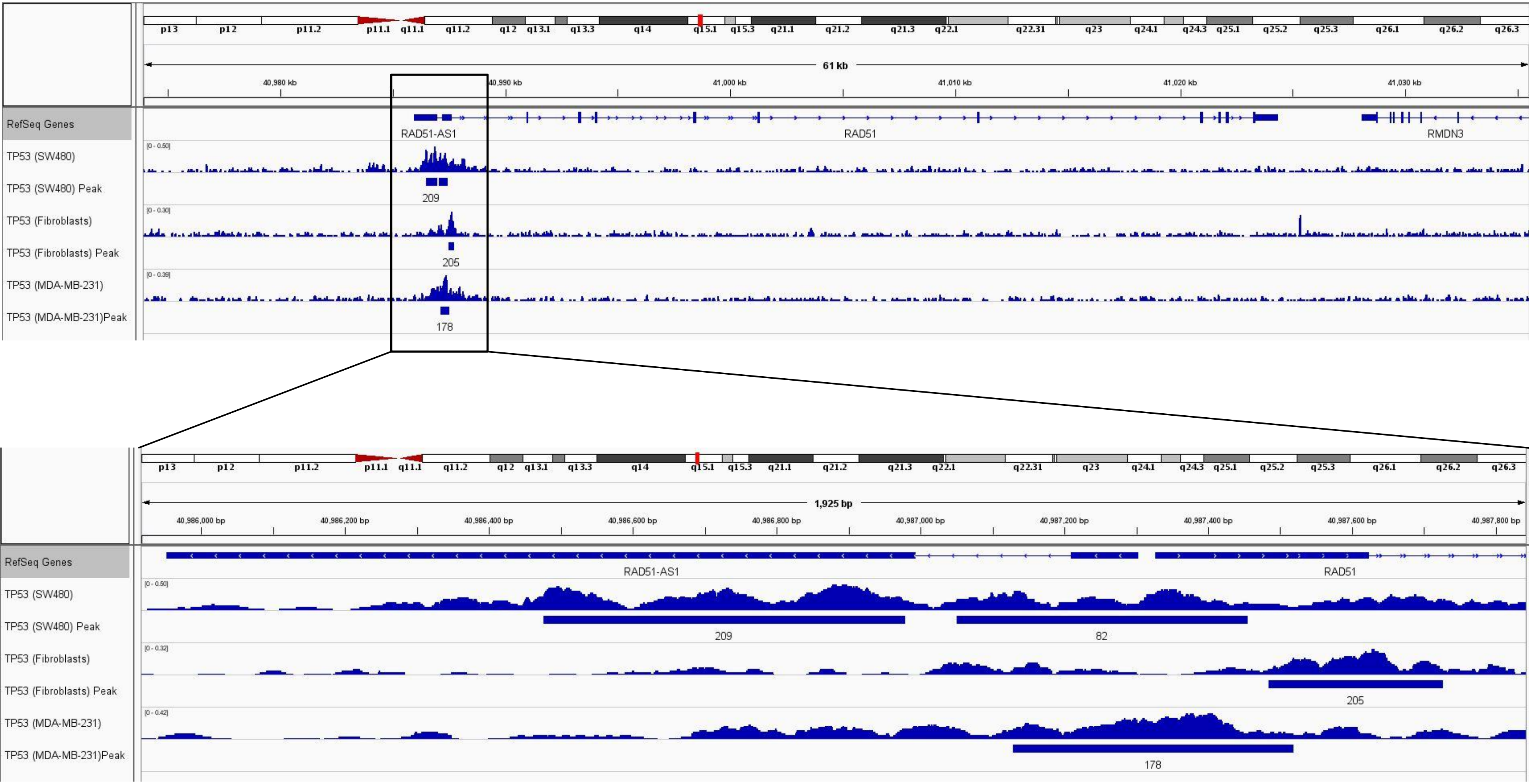

Sup. Figure 2 for Figure 6
ChiP-Seq analysis show that p53 has binding sites on RAD52 promoter region.

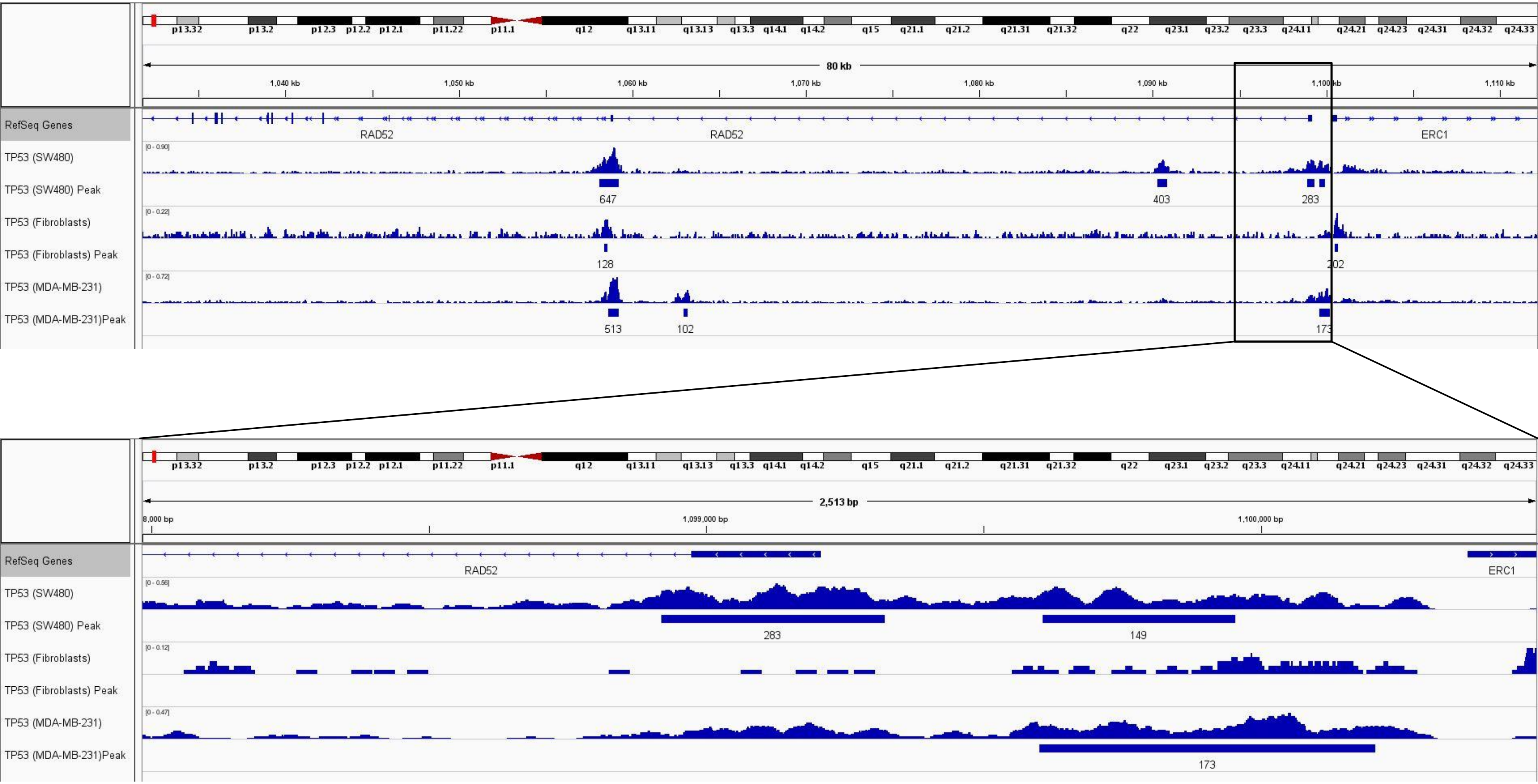

Figure 1 D

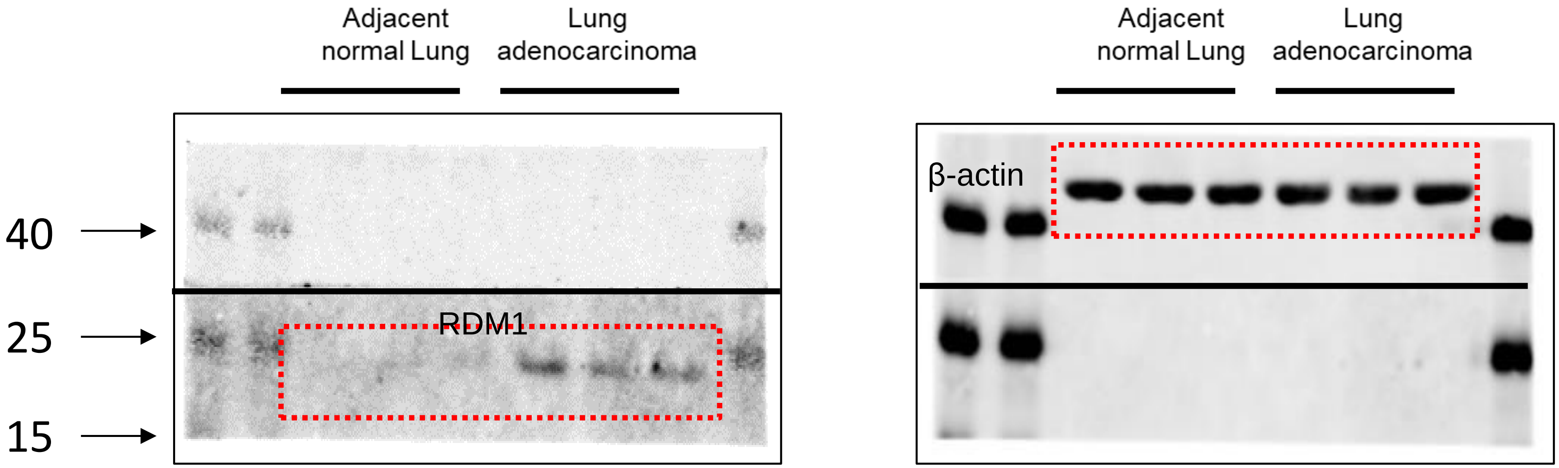

Original western blots or whole gels for the images shown in **Figure 1 D**
. The cropped images are highlighted in the red lines.

Figure 3 A

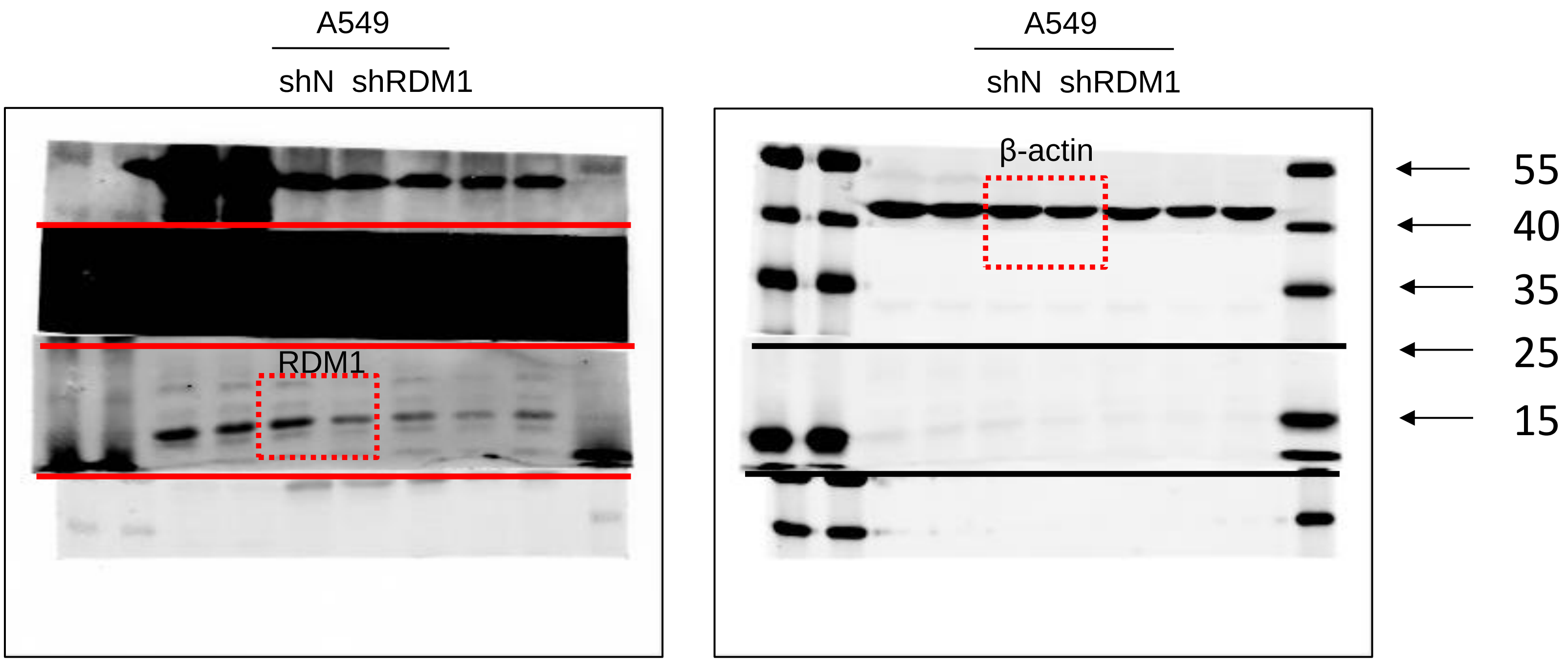

Original western blots or whole gels for the images shown in **Figure 3 A**
. The cropped images are highlighted in the red lines.

Figure 5 A

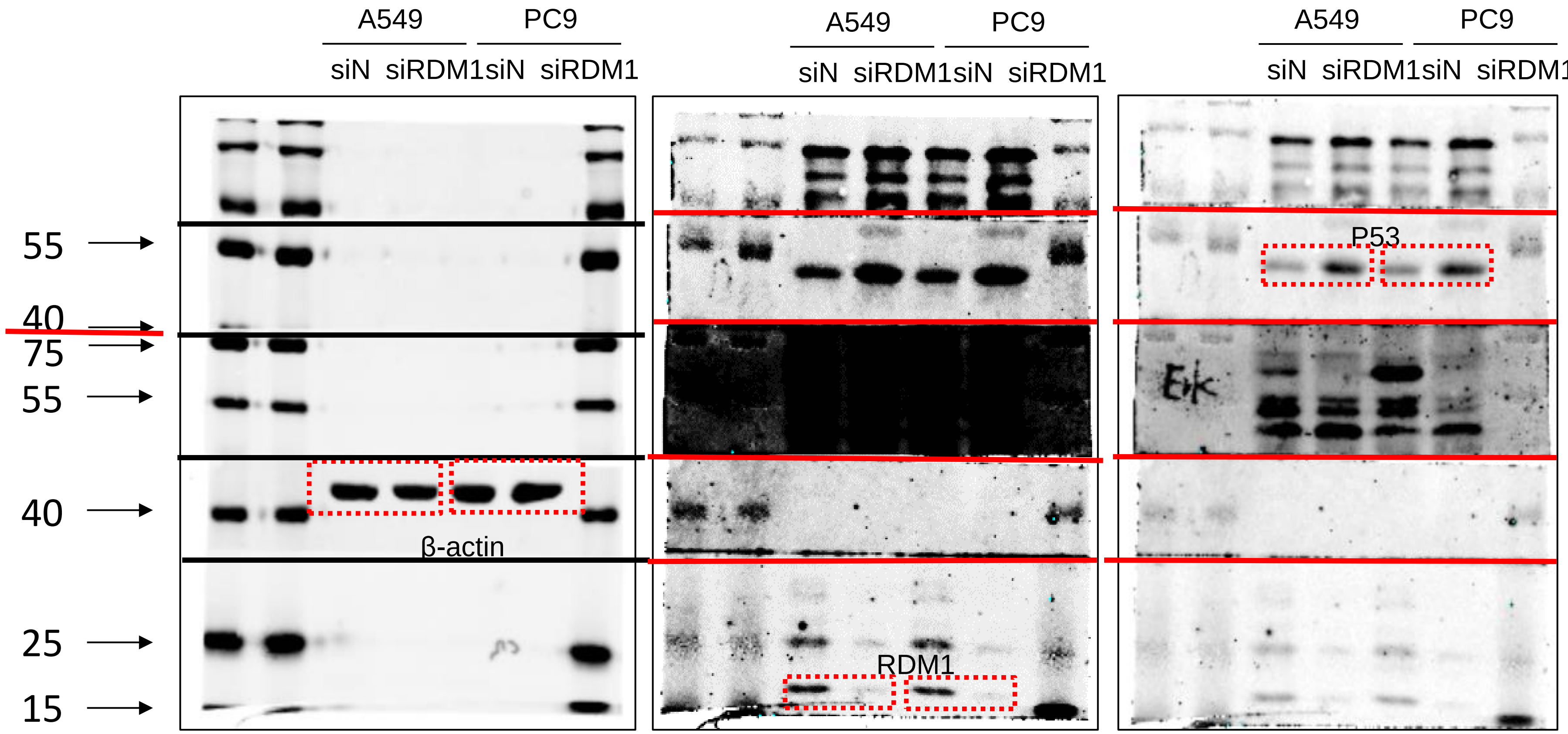

Original western blots or whole gels for the images shown in **Figure 5 A**
. The cropped images are highlighted in the red lines.

Figure 5 C

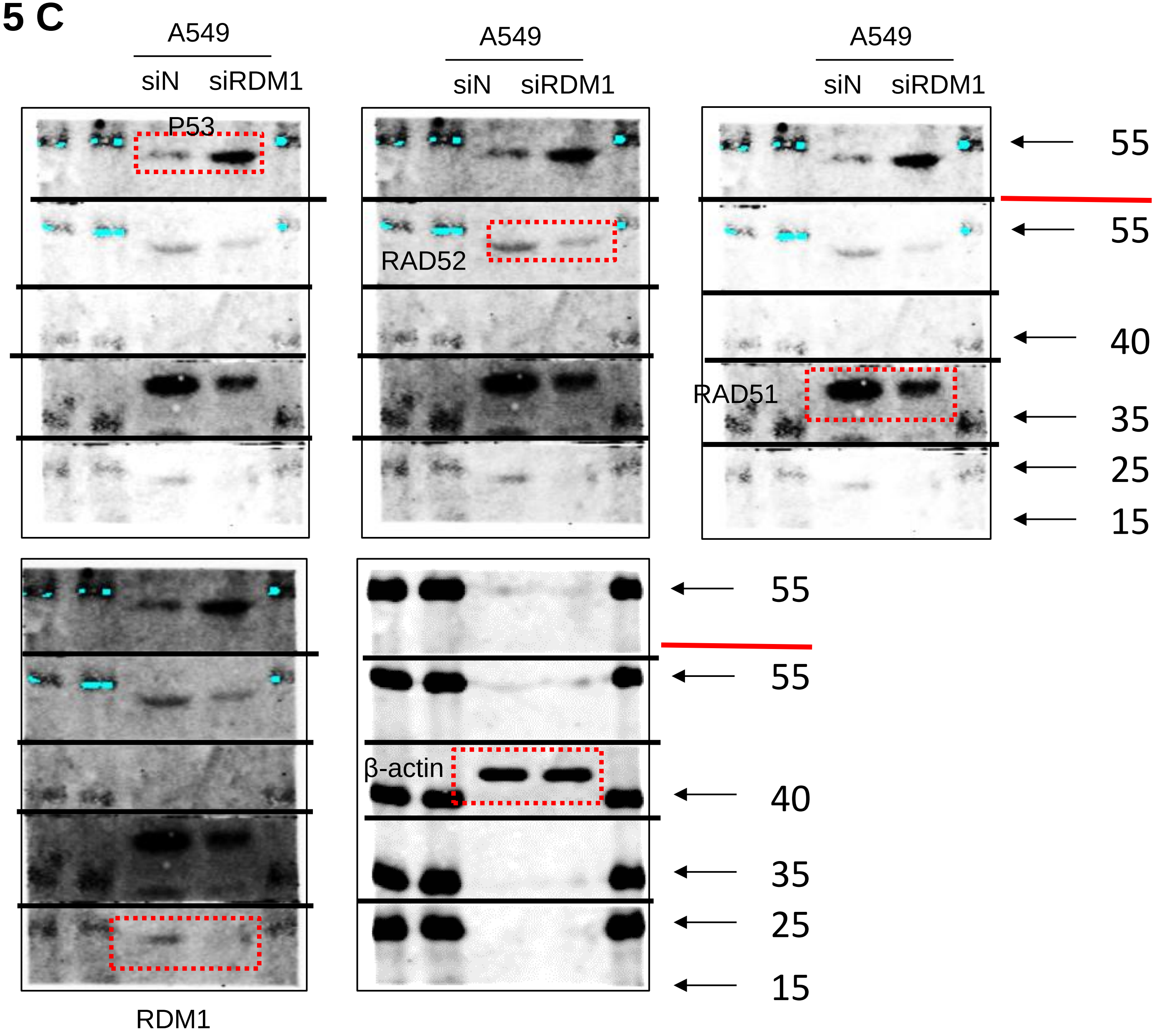

Original western blots or whole gels for the images shown in **Figure 5 C**
. The cropped images are highlighted in the red lines.

Figure 5 D

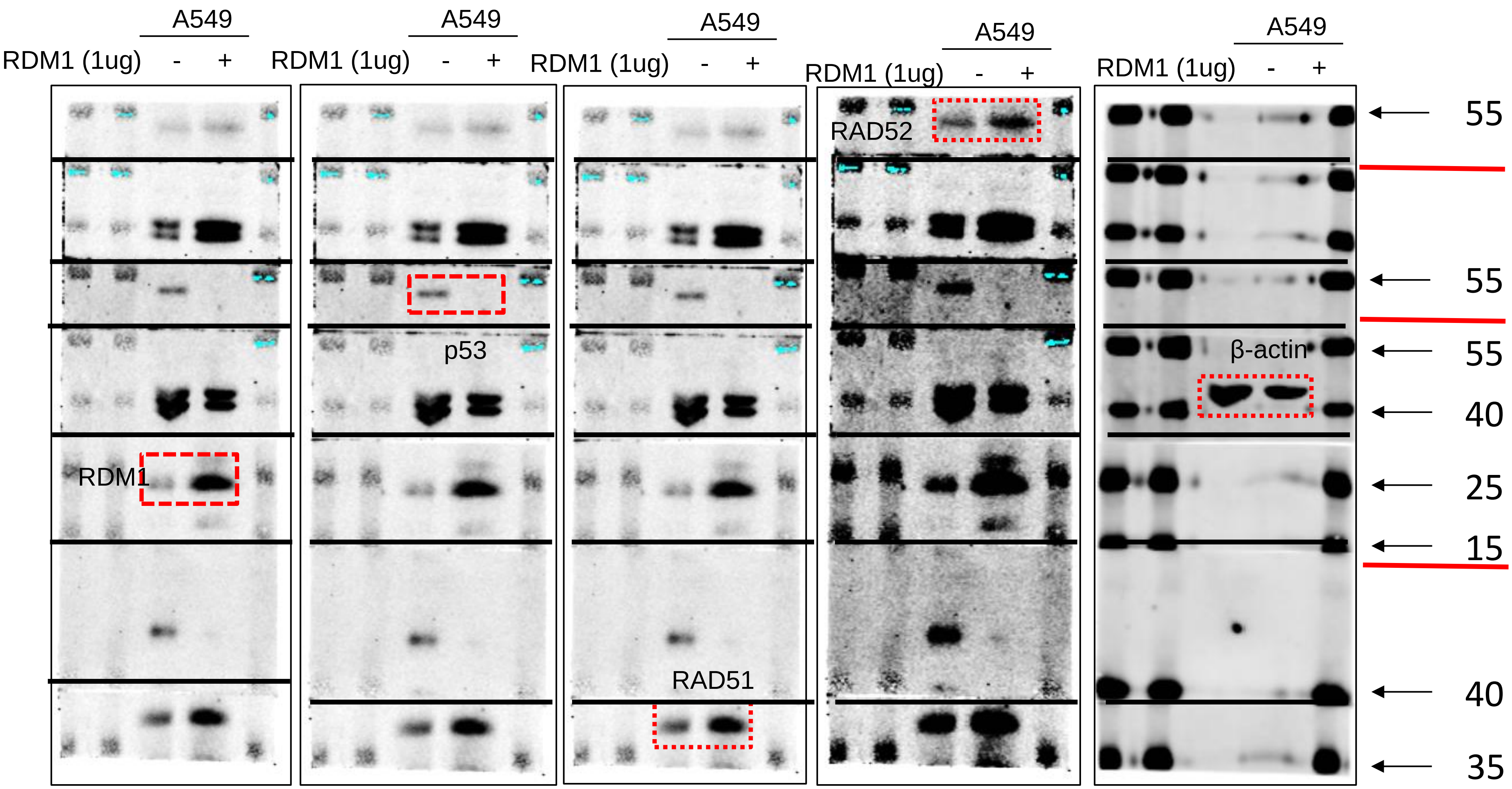

Original western blots or whole gels for the images shown in **Figure 5 D**. The cropped images are highlighted in the red lines.

Figure 6A

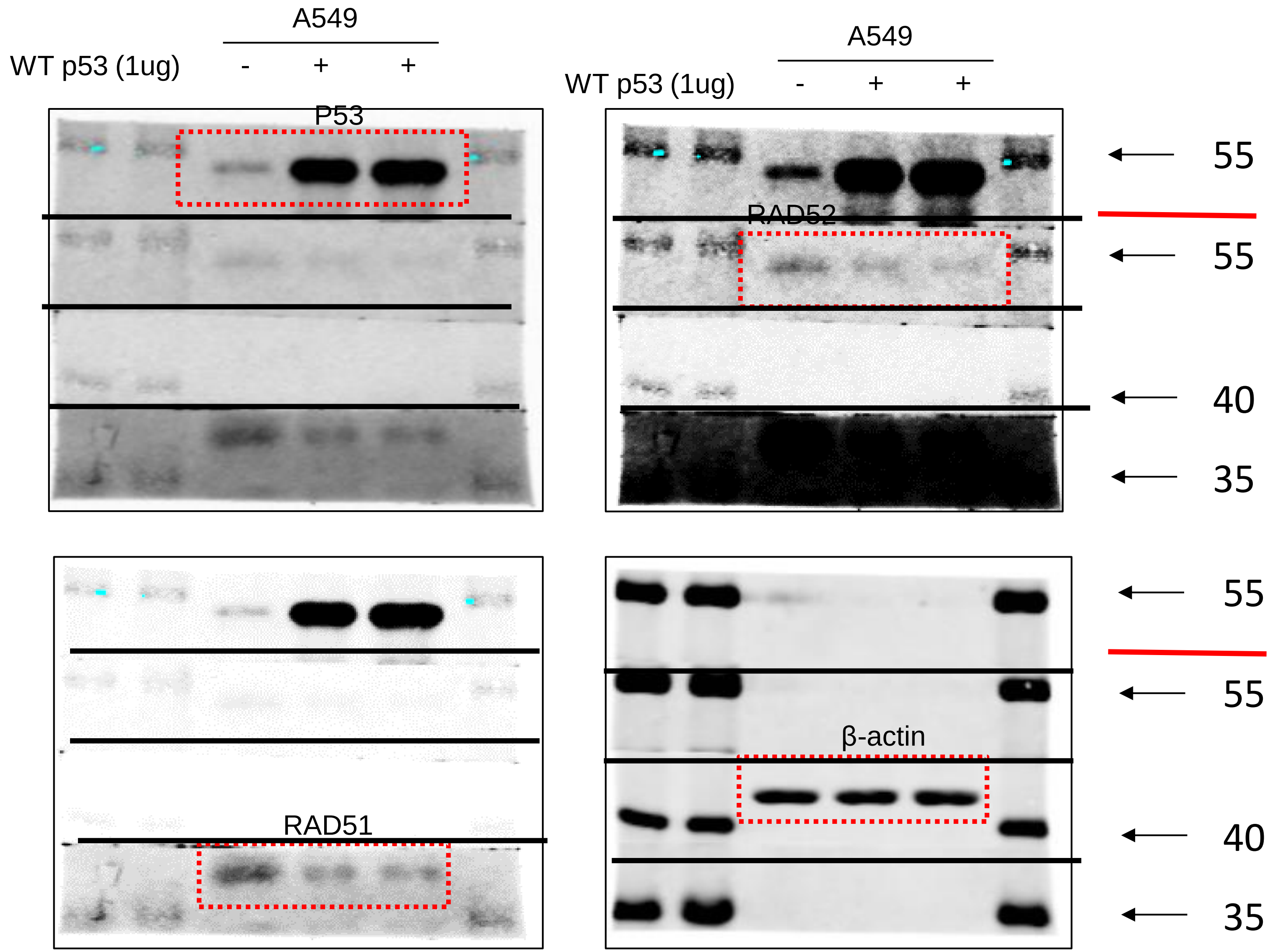

Original western blots or whole gels for the images shown in **Figure 6 A**. The cropped images are highlighted in the red lines.

Figure 7A

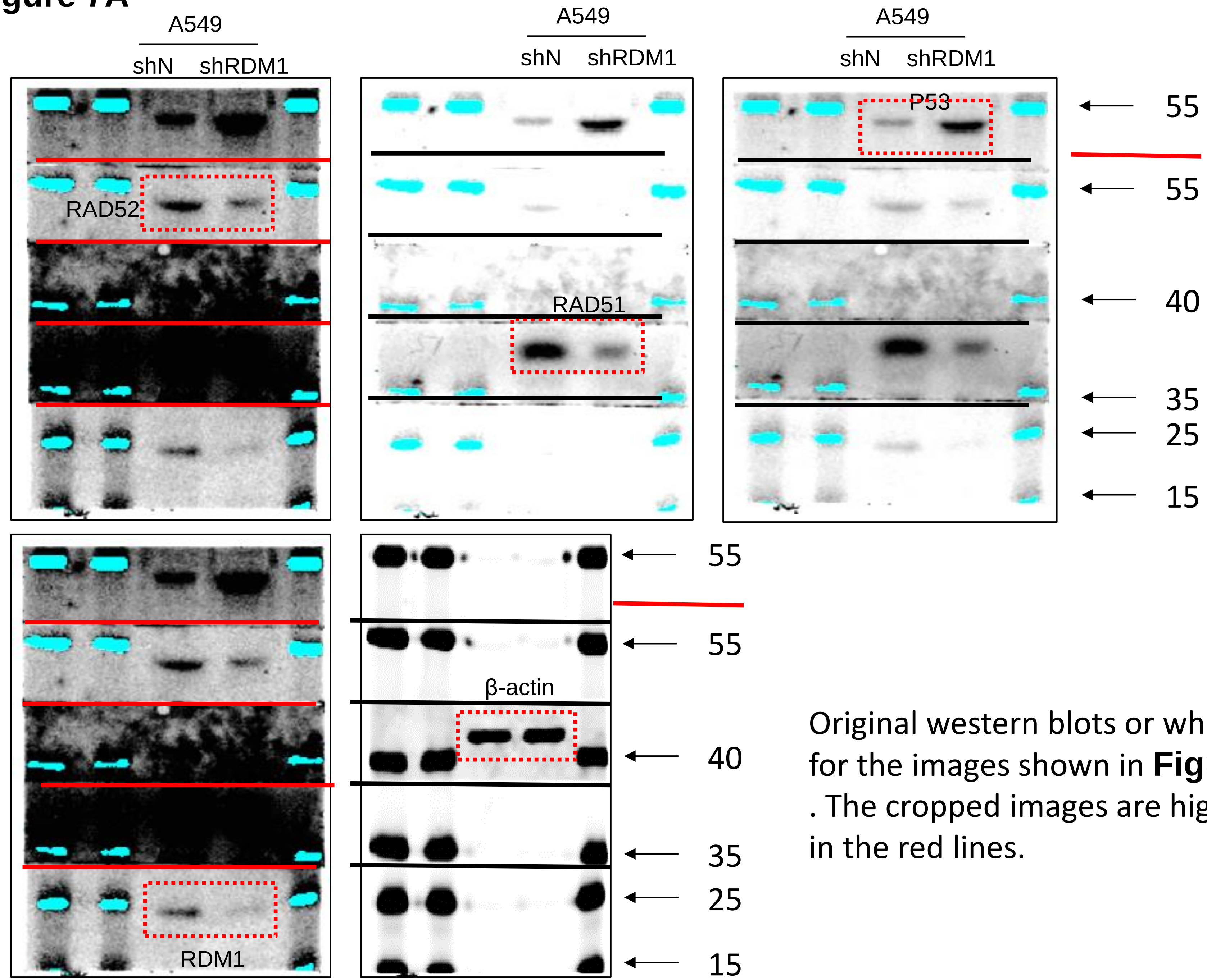

Original western blots or whole gels for the images shown in **Figure 7 A**. The cropped images are highlighted in the red lines.

Figure 7C

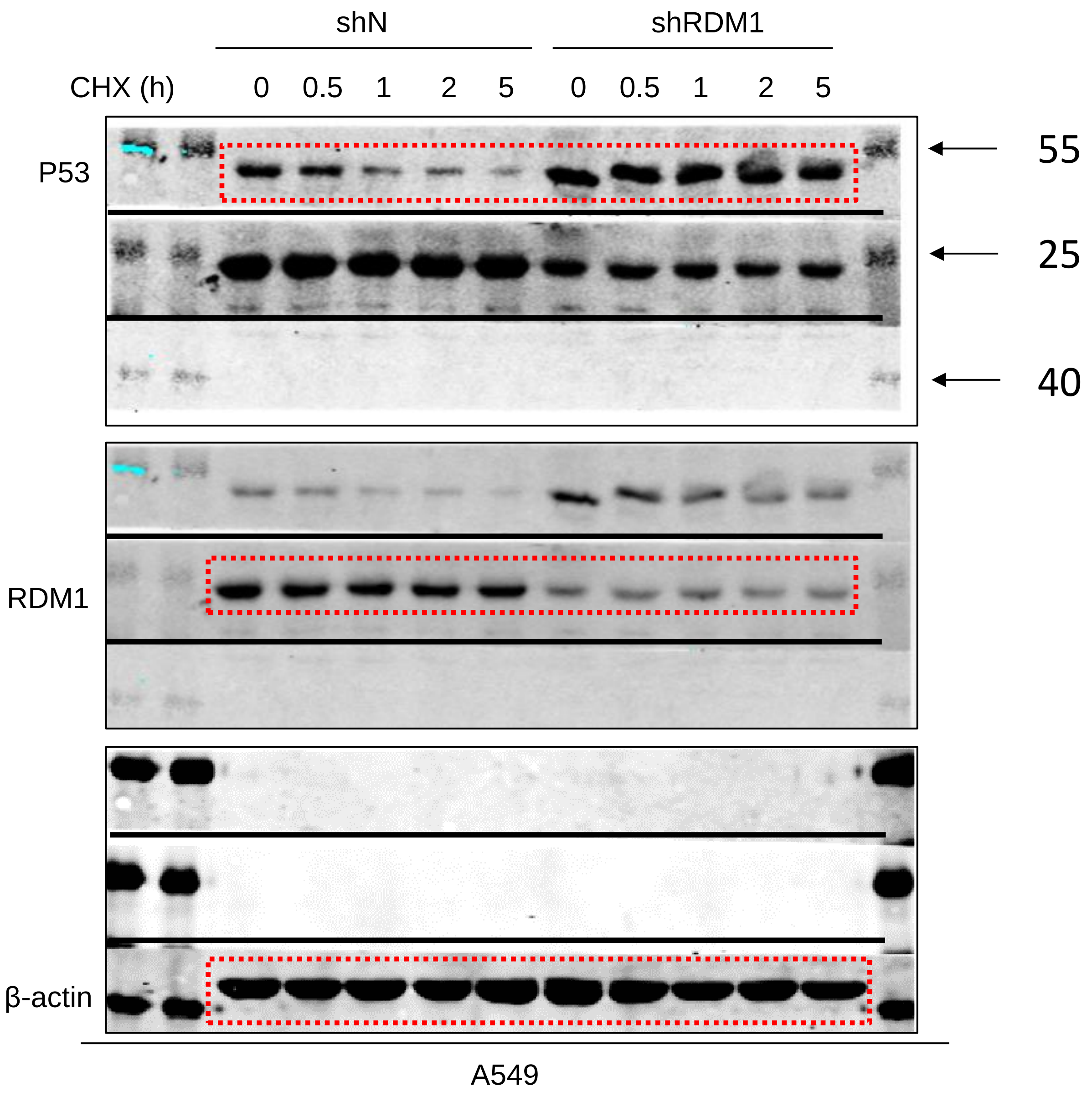

Original western blots or whole gels for the images shown in **Figure 7 C**. The cropped images are highlighted in the red lines.
